# Aspirin Use among Saudi Adults: The Prevalence and Users’ Characteristics

**DOI:** 10.1016/j.jsps.2022.02.003

**Published:** 2022-02-11

**Authors:** Sultan Alghadeer, Abdulrahman M. Alwhaibi, Abdulaziz Alhossan, Salmeen D. Babelghaith, Abdullah M. Mubarak, Sana Samreen, Nouf N. Alameel, Noura N. Aljabali, Mohamed N. Al-Arifi

**Affiliations:** a Department of Clinical Pharmacy, College of Pharmacy, King Saud University, Riyadh, Saudi Arabia; b Department of Basic Sciences, PSCEMS, King Saud University, Riyadh, Saudi Arabia; c Volunteering Researcher in the Drug and Poison Information Center (DPIC), Clinical Pharmacy Department, College of Pharmacy, King Saud University

**Keywords:** Prevalence, Characteristics, Aspirin, Saudi Arabia

## Abstract

**Introduction:**

Despite the public routine use of aspirin as cardio-prophylaxis agent, its use is only recommended in particular situations, and not as usual primary prevention. Only few local studies investigate the use of aspirin in patients with certain diseases, but not within the public population. The purpose of this study was to evaluate the prevalence of aspirin use and identify the demographic and clinical characteristics among Saudi users.

**Methodology:**

A cross-sectional study targeting Saudi adults in Saudi Arabia was conducted over a period of four months in 2021 using online Google forms. The study collected data to assess the prevalence of use, use of aspirin according to prevention type, users’ characteristics and comorbidities. Additionally, a self-assessment of knowledge, perception, reasons and attitude towards aspirin use among Saudi adults was conducted. A chi-square test was used to determine the association between the variables. A P-value ≤ 0.05 was considered statistically significant.

**Results:**

The prevalence of aspirin use was 47%. Regarding the self-assessed aspirin knowledge, the majority of the respondents (n = 481; 62.4 %) found to have good knowledge. Less than half of the participants (n = 341; 44%) use aspirin as primary prevention agent while only 23 participants (2.9%) use aspirin as secondary prevention agent. There was a significant difference between gender and user type (p = 0.001). With regards to comorbidities, hypertension, hyperlipidemia, diabetes, and obesity were common among the primary users of aspirin. Significant associations were found (p = 0.001) between participant’s user type and the following characteristics such as smoking status, past medical history, presence of comorbidities.

**Conclusion:**

Aspirin use is commonly prevalent Saudi population with good level of knowledge of the therapy; however, its popular use as primary preventive agent for CVD may necessitate medical advice based on the level of cardiovascular risk.

## 1 Introduction

Aspirin has been approved by the U.S. food and drug administration (FDA) to be use as secondary prevention agent of cardiovascular diseases (CVD) (Durlaza-prescribing information, 2015). Its advantage was significantly noticeable in decreasing CVD morbidity and mortality in patients with high risk of CVD or those underwent CVD procedures (Bryant, et al., 2006, Algra and Rothwell, 2012, Williams et al., 2015, Alkhail et al., 2016, Abdelaziz et al., 2019). Several randomized clinical controlled trials have assessed the role of aspirin as primary prevention agent. Although early clinical trials have showed advantages of aspirin in decreasing CV events, more new trials have contradictory results with even an indicator towards net harm (Mahmoud et al., 2019, Batais et al., 2021). Additionally, the evidence base of aspirin’s safety and efficacy for primary prevention vary amongst randomized controlled trials (de Gaetano, 2001, Ridker et al., 2005, Ogawa et al., 2008, Fowkes et al., 2010, Abdelaziz et al., 2019) making important variability in the professional guidelines (Pignone et al., 2010, Goldstein et al., 2011, Abdelaziz et al., 2019, Bibbins-Domingo, 2016). Such difference makes the guidelines of American Heart Association/ American College of Cardiology support the personalized intervention of aspirin as primary prevention agent based on particular patient’s factors (Arnett Donna et al., 2019).

Aspirin is suggested for modestly high-risk patients by the U.S. Preventive Services Task Force (Williams et al., 2015), but it is not suggested for primary prevention for any risk level by the European Society of Cardiology (Bryant et al., 2006). Also, the U.S. FDA doesn’t recommend routine use of aspirin for primary prevention, but it’s stated it might be suitable under recommendation of healthcare provider to higher-risk people (Williams et al., 2015). Additionally, the role of aspirin as primary prevention of cancer is still controversial, and it may be more helpful in some types of cancer (Algra and Rothwell, 2012, van Kruijsdijk et al., 2015, Haykal et al., 2019). The recent *meta*-analysis of randomized controlled trials has not found any significant decreases in mortality of cancer or cancer occurrence when compared aspirin use with placebo. This study reported that the use of aspirin for primary prevention of cancer can increase the risk of bleeding likened to placebo or no aspirin (Haykal et al., 2019).

Around of 23.4% (≈ 29 million participants) of adults in the United States use aspirin as primary prevention agent for CVD. Of those (22.8%; 6.6 million) use aspirin independently without any medical advice (O'Brien et al., 2019). In Saudi Arabia, few studies have been published to find the prevalence of aspirin use. Most of these studies were published among diabetic patients. A study aimed to determine the prevalence of aspirin use among type 2 diabetic patients and evaluate the concordance in aspirin use as recommended by the Aspirin-Guide application was recently conducted. The study found high prevalence of aspirin uses among these patients. Twenty-six percent of those patients were inappropriately on aspirin according to the Aspirin-Guide application recommendations whereas 37.7% of the patients who should be on aspirin were not taking aspirin (Batais et al., 2021). Similar study was done at different region of Saudi Arabia to evaluate the recommendation of aspirin as well as statin treatment in patients with diabetes to prevent CVD. This study found that aspirin was indicated and prescribed in 36.2% of the patients while in aspirin was indicated but not recommended in 17.0% of the patients (Alkhail et al., 2016).

Only few local studies investigate the use of aspirin in patients with certain diseases, but not within the public population. Additionally, presence of public data regarding the aspirin usage would assess the clinical compliance of our patients and clinicians practice toward the national guidelines and recommendations. Therefore, the purpose of this study was to evaluate the prevalence of aspirin use and identify the demographic and clinical characteristics among Saudi users.

## 2 Method

### 2.1 Study design and participants

A cross-sectional online-based study was conducted among adult Saudi people for over a period of four months in 2021 using self-administrated questionnaires. All adult Saudi participants were eligible for our study. Participants who were below 18 years old or non-Saudis were excluded from the study. The health section of Institutional Review Board at King Saud University, Saudi Arabia provided the ethical approval (No. E-20–4625) for this study.

### 2.2 Study questionnaires and procedure

An anonymous research survey was prepared to estimate the prevalence of aspirin use and identify the demographic and clinical characteristics of Saudi users. A self-administered validated questionnaire was adapted from the previous studies published in this regard (Williams et al, 2015). The study questionnaire included demographic details of the respondents like age, gender, job, educational level, past medical history and a question about the place of or region of living in Saudi Arabia. In addition to determine the prevalence of aspirin use and participant’s characteristics according to prevention type, the survey revealed the participant’s knowledge and perception of aspirin, and showed attitude, pattern and reason use of aspirin among primary and secondary prevention users of aspirin. The study questionnaire was evaluated by a group of clinical professors and researchers from college of pharmacy at King Saud University with substantial experience in survey design after forward/backward translation into Arabic Language. A pilot study was conducted before proceeding with the original study with a sample of 10 randomly selected participants. The pilot study was conducted to ensure the readability and accuracy of the questionnaire among the targeted population. According to the results of the pilot study, the research questionnaires were revised. The results of the pilot study were not included in the research. The reliability of the questionnaire was assessed with Cronbach’s alpha coefficient of 0.7. All Saudi adults were enrolled using different social media. An invitation link containing a survey questionnaire was sent to the participants without any previous measures. For the data collection, we used the snowball technique where any person recruited to do the survey provides multiple referrals.

### 2.3 Data analysis

The data were extracted and were analyzed using the Statistical Package for Social Sciences version 26.0 (SPSS Inc., Chicago, IL, USA). A p-value of less than 0.05 was considered statistically significant. Descriptive analysis was performed. Categorical data were calculated as frequencies and percentages. Other statistical tests like the chi-square test were applied to find out the association between the variables.

## 3 Data management

**Fig. 1:**
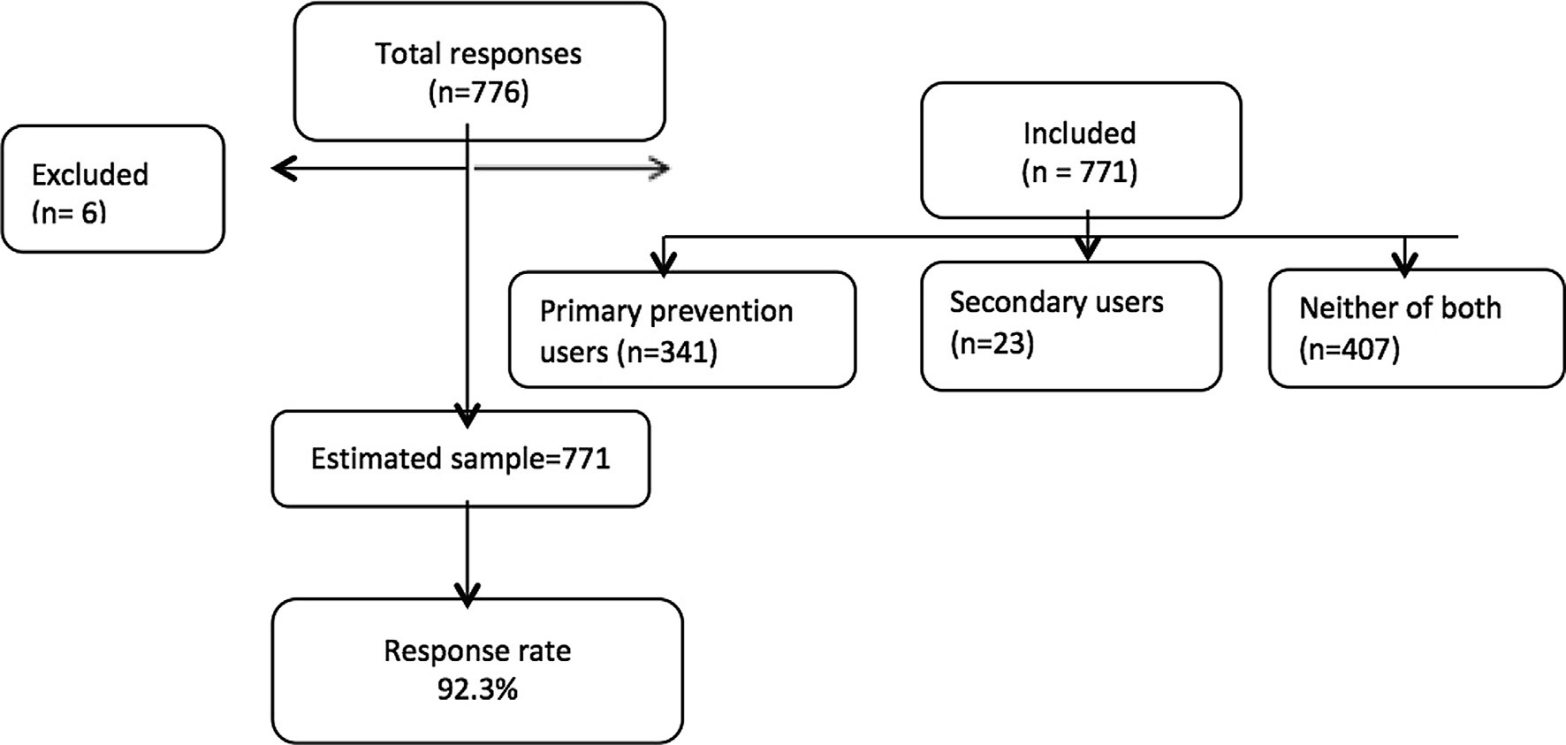
Flowchart of responses.

Data extraction process is a part of research which involves a thorough assessment of both completed and incomplete questionnaires (Osborne, 2013). The accuracy and completeness of the data were examined in this study. The study excluded questionnaires if there had been any missing or incomplete responses (Fig. 1).

## 4 Results

A total of 776 respondents had received the questionnaire. Among the respondents, 771 completed the questionnaire with a response rate of 92.3%. Female were the predominant participants (n = 555; 72%). The majority of the study participants (n = 329; 42.7%) were aged less than 45 years. Among all participants, most of them were university graduates (67.1%), and 37.5%, 28.4%, and 21.7% were employed, non-employed, and retired respectively. The majority of our subjects live in the central region of Saudi Arabia (71.6%). Regarding the healthy habits, about 43.2% of the individuals “do physical activity”, and 82.9% were nonsmokers. More than half of the respondents (56.2%) did not have any previous medical history. For those who have comorbidities, hyperlipidemia was the highest comorbidity (n = 157; 20.4%), followed by hypertension (n = 147; 19.1%), diabetes (n = 132; 17.1 %), and obesity (n = 125; 16.2%). The vast majority of respondents (97.7%) did not have any cancer history. The demographic characteristics of the respondents in the study sample are shown in Table 1

**Table 1:** Baseline participant’s characteristics (n = 771).

Variables	N	%
**Gender**		
Male	216	28.0
Female	555	72.0
**Age**		
Less than 45 years	329	42.7
45–55	261	33.9
56–65	136	17.6
More than 65 years	45	5.8
**Education level**		
Non	7	0.9
Primary /secondary school	35	4.5
High school	140	18.2
University	517	67.1
Postgraduate	72	9.3
**Insurance**		
Governmental hospital	222	28.8
Private	270	35.0
Non	279	36.2
**Smoking**		
Current	75	9.7
Former	57	7.4
Non-smoker	639	82.9
**Exercise**		
Yes	333	43.2
No	438	56.8
**Exercise time**		
30 mints daily	123	36.9
30 mints once weekly	54	16.2
30 mints twice weekly	52	15.6
30 mints three-time weekly	56	16.8
30 mints four-time weekly	48	14.4
**Any past medical history disease**		
Yes	338	43.8
No	433	56.2
**Comorbidities**		
Diabetes	132	17.1
Hypertension	147	19.1
Obesity	125	16.2
Stroke	5	0.60
Hyperlipidemia	157	20.4
Angina	7	0.90
Heart attack	9	1.20
**History of cancer**		
Yes	18	2.3
No	753	97.7
**Cancer type**		
Breast cancer	7	0.9
Colon	1	0.1
Bladder	1	0.1
Others	9	1.2

*Missing data.

Regarding self-assessment of aspirin knowledge, the majority of the respondents (n = 481; 62.4 %) had good knowledge, and 14.4% of them found to have excellent knowledge of Aspirin. Among the participants, one-third of them (n = 266; 34.5%) agreed that aspirin use benefits them. Slightly more than half (n = 393; 51%) of our subjects agreed that aspirin is effective to reduce stroke. Detailed of participant’s knowledge and perception of aspirin are shown in Table 2.

**Table 2:** Participants’ knowledge and perception of aspirin.

**Aspirin knowledge (self-assessed)**	N	%
Excellent	111	14.4
Good	481	62.4
I don’t know	179	23.2
**Agreement with benefit of aspirin**		
Strongly agree	77	10
Agree	266	34.5
Neutral	337	43.7
Disagree	74	9.6
Strongly disagree	17	2.2
**Aspirin efficacy to reduce stroke**		
Effective	393	51.0
Not effective	32	4.2
I don’t know	346	44.9

**Fig. 2:**
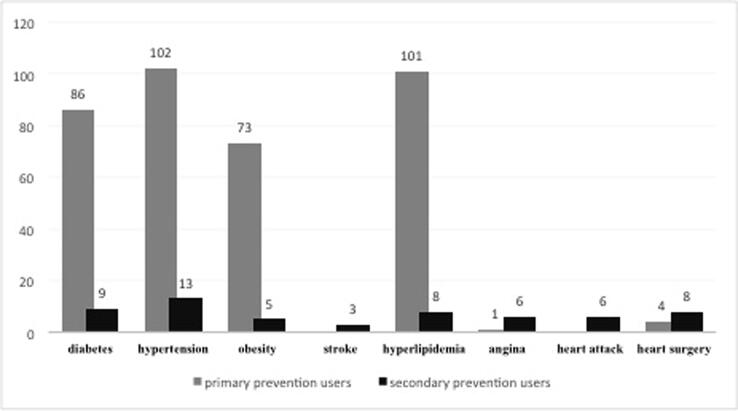
Comorbidities among the primary and secondary prevention users of aspirin.

The prevalence of aspirin use among the Saudi population was 47%. According to the use of aspirin based on the type of prevention, slightly less than half (n = 341; 44%) were belonged to primary prevention users while (n = 23; 3%) were secondary prevention users. There was a significant difference between gender and user type (p = 0.001). Females were higher as primary prevention users compared to males (66% vs. 34%) while males used aspirin as secondary prevention agent more than females (78.3% vs. 21.7%). Table 3 shows the different participant’s characteristics according to prevention type. The results found a significant association between participant’s user type and participant’s smoking status, past medical history, and presence of comorbidities (p = 0.001). With regards to comorbidities, hypertension, hyperlipidemia, diabetes, and obesity were common among primary users of aspirin. (Fig. 2).

**Table 3:** Participants’ characteristics according to prevention type (n = 364).

**Characteristics**	**Primary prevention**	**Secondary prevention (n = 23)**	**P value**
	**(n = 341)**		
**Gender**			
Male	116(34)	18(78.3)	0.001
Female	225(66)	5(21.7)	
		
**Age groups (years**)			
Less than 45 years	109 (32)	5(21.7)	0.279*
45–55	120(35.2)	6(26.1)	
56–65	80(23.5)	9(39.1)	
More than 65 years	32(9.4)	3(13.0)	
Region*			
Central	266(78.0)	10(43.5)	0.001*
Eastern	39(11.4)	8(34.8)	
Western	15(4.4)	4(17.4)	
North	11(3.2)	1(4.3)	
South	7(2.1)	----	
**Job**			
Employer	125(36.7)	6(26.1)	0.015*
Non-employer	93(27.3)	3(13.0)	
Students	7(2.1)	–	
Retired	93(27.3)	9(39.1)	
Businessman	12(3.5)	5(21.7)	
Others	8(2.3)	–	
**Education level**			
Non	6(1.8)	–	0.305*
Primary/secondary school	20(5.9)	2(8.7)	
High school	62(18.2)	4(17.4)	
University	221(64.8)	12(52.2)	
Postgraduate	32(9.4)	5(21.7)	
**Insurance**			
Governmental hospital	114(33.4)	4(17.4)	
Private			236
Non	116(34.0)	11(47.8)	
	111(32.6)	8(34.8)	
**Smoking**			
Current	43(12.6)	5(21.7)	
Former	26(7.6)	10(43.5)	0.001*
Non-smoker	272(79.8)	8(34.8)	
**Exercise**			
Yes	145(42.5)	11(47.8)	0.667
No	196(57.5)	12(52.2)	
**Any past medical disease**	1		
Yes	98(58.1)	23(1 0 0)	0.001
No	143(41.9)	–	
**Comorbidities**			
Diabetes	86(25.2)	9(39.1)	0.218
Hypertension	102(29.9)	13(56.5)	0.025*
Obesity	73(21.4)	5(21.7)	1.00*
Stroke	–	3(13.0)	0.001*
Hyperlipidemia	101(29.6)	8(34.8)	0.632
Angina	1(0.3)	6(26.1)	0.001*
Heart attack	–	6(26.1)	0.001*
Heart surgery	4(1.2)	8(34.8)	0.001*
**Cancer**			
Breast cancer	2(0.6)	–	1.00*
Conon	1(0.3)	–	
Bladder	1(0.3)	–	
Others	5(1.5)	1(4.3)	
		

* Fisher’s exact test.

Table 4 depicts the participant’s attitude, pattern, and reasons for the use of aspirin. The advice from healthcare providers (55.1% and 60.9% in primary and secondary prevention users respectively) was the most common reason to use aspirin followed by the advice from healthcare providers at primary care (19.6% and 30.4% in primary and secondary prevention users respectively). Most of secondary prevention users are currently taking prescribed aspirin (78.3% vs. 34.3) while most of primary prevention users are either previously used aspirin with prescription (34% vs. 17.4%) or currently using aspirin without prescription (31.7% vs. 4.3%). Regarding the pattern/type of aspirin use, the current use of aspirin on regular basis was higher among the secondary prevention users compared to the primary prevention users (78.3% vs. 34.3%) while the previous and lack of regular use of aspirin were seen more among the primary prevention users (Table 4). When the primary prevention users asked about the indications of aspirin, about 71 %, 27%, and 21.7% reported using aspirin for clots prevention, pain relief and heart attack prevention respectively. Despite of the majority of the primary prevention users had a history of GI or stomach bleeding (n = 336; 98.5%), approximately 65.1% of the primary prevention users don’t discuss the regular use of aspirin with medical staff. The results found a significant association between pattern/type of aspirin use, use of aspirin by prescription, and heart attack prevention.

**Table 4:** Attitude, patterns and reasons among primary and secondary prevention users of aspirin (n = 364).

**Characteristic**	**Primary prevention (n = 341)**	**Secondary prevention (n = 23)**	**P value**
**Use aspirin advise from**			
Health care providers	188(55.1)	14(60.9)	0.276
Medical staff at primary care/ provider’s office	67(19.6)	7(30.4)	
Friends	57(16.7)	1(4.3)	
I don’t know	29(8.5)	1(4.3)	
**Use aspirin by prescription**			
Yes, currently use the prescription aspirin	117(34.3)	18(78.3)	0.001
Yes, previously used aspirin with prescription	116(34.0)	4(17.4)	
No, currently using aspirin without a prescription	108(31.7)	1(4.3)	
**Pattern/type of aspirin use**			
Currently taking aspirin on a	85(24.9)	17(73.9)	0.001
regular basis^¶^			
Previously took aspirin on a	136(39.9)	5(21.7)	
regular basis^¶^			
never took aspirin on a regular basis^¶^	120(35.2)	1(4.3)	
**Indication of aspirin (yes)**			
Heart attack prevention	74(21.7)	14(60.9)	0.001
Stroke prevention	46(13.5)	6(26.1)	0.118*
Clots prevention	242(71.0)	17(73.9)	0.818
Relieve pain	92(27.0)	2(8.7)	0.081
Cancer prevention	10(2.9)	1(4.3)	0.517*
Alzheimer prevention	10(2.9)	–	1.0*
**Discussed regular aspirin use with medical staff**			
Yes	119(34.9)	9(39.1)	0.822
No	222(65.1)	14(60.9)	
**History of GI or stomach bleeding**			
Yes	336(98.5)	2(8.7)	0.066*
No	-----	21(91.3)	

Regular basis: daily

* Fisher’s exact test.

## 5 Discussion

This research showed the prevalence and the associated characteristics for using aspirin among Saudi population. Although the prevalence of aspirin use may differ from one study to another that may be influenced by several factors including the study method, types of respondents, and demographics of the subjects, it is evidenced that public utilization of aspirin is common, particularly for the primary and secondary prevention of CVD (Webster et al., 2009). It was found that the prevalence of aspirin use was 30% of cardiovascular patients and 41% among the general community in the United States (Zhou et al., 2014, Stuntz and Bernstein, 2016). Also in the United States, around 41% of older adults (≥40 years old) use aspirin, and about 43% of population use aspirin for the prevention of CVD (Pignone et al., 2007, Van't Hof et al., 2017). Additionally, a national survey conducted by Williams et al among 2,509 American adults showed that the prevalence of aspirin use is 52% with about 81% and 12% of them use aspirin as primary and secondary preventive agent respectively (Williams et al., 2015). The abovementioned results seem consistent with our findings that reported that 47% of Saudi participants are using aspirin. Most of those Saudi users of aspirin were older than 45 years old (69%) and use their aspirin as primary prevention agent (94%). In Contrast, the prevalence of aspirin use in Europe is very low. The prevalence of aspirin use found to be 10% among Italians for primary or secondary prevention of CVD, and 3% among Switzerland population for primary prevention of CVD (Rodondi et al., 2008, Lugo et al., 2014). Such differences were explained by the hesitancy of physicians in Europe to prescribe aspirin as primary prevention agent and the weak of “direct-to-consumer advertising” that is strongly available in other countries (Rodondi et al., 2008).

With regards to comorbidities and characteristics of aspirin users, hypertension (29.9%) followed by hyperlipidemia (29.6%), diabetes (25.2%), and obesity (21.4%) were the most prevalent conditions of comorbidities reported among primary prevention users of aspirin in our study. By comparing these results to the U.S. national survey, incidence of hypertension (44%) and hyperlipidemia (44%) among primary prevention users were higher while the incidence of diabetes (16%) was lower (Williams et al., 2015). While among the aspirin users in Italy, the presence of diabetes (52.0%), hypertension (42.6%), and hyperlipidemia (38.6%) were higher. Approximately 336 (98.5%) of our participants who used aspirin as primary prevention agent have history of gastrointestinal bleeding. Despite the self-reported and clarity of question about the gastrointestinal bleeding, such high incidence of bleeding could be exaggerated and go beyond the global data and contravene safety measure of using aspirin. The gastrointestinal bleeding among aspirin users’ in the United States was found to be 53% only (Williams et al., 2015). Additionally in a total of 5725 of aspirin users’, around 713 cases of gastrointestinal bleeding could be avoided if aspirin used appropriately (Rodondi et al., 2008). Also, it’s estimated that there is one new case of gastrointestinal bleeding for each 3333 aspirin users’ (Rose et al., 2011). As result of our high reporting of gastrointestinal bleeding that contradict appropriate use of aspirin and inconsistent of aspirin users’ globally, further local research focused on the prevalence and incidence of gastrointestinal bleeding among aspirin users may become necessary.

Our findings observed that use of aspirin is significantly associated with gender and user type, social habits and presence of disease. Our results were somewhat similar to previous study results that reported that healthy lifestyle, including trying to stop smoking, eating healthy food, and getting more exercise were significantly associated with aspirin use (Williams et al., 2015). Furthermore, Zhou et al reported that increased use of aspirin were consistent with participant’s age, sex, race, and selected medical conditions, including CVD, arthritis, peptic ulcers, cancer, and severe headache (Zhou et al., 2014). In our study, aspirin use was not significantly associated with the any type of cancer; however, the use was significantly associated with presence of hypertension, stroke, angina, heart attack and heart surgery.

With the public global increase of aspirin usage based on the literatures, concerned studies raised a question whether such use come from medical advice or not (Luepker at al., 2015). About 55.1% use aspirin as primary preventive agent based on “healthcare advice” while only 31.7% and 34.9% of our subjects who used aspirin as primary prevention agent is “currently using aspirin without a prescription” and “discussed regular use of aspirin with medical staff”. Among the aspirin users for primary prevention of CVD in the U.S., the use of aspirin based on “healthcare advice” was almost similar (50%) to our findings while population in the U.S. held more (51%) discussion with medical staff regarding the regular aspirin usage (Williams et al., 2015). However, both participants in our study and in the national U.S. survey showed exactly similar results (both 77%) regarding the knowledge of aspirin therapy. About 67.1% of our participants were university graduates. This result is very consistent with the Saudi’s gross enrollment ratio in tertiary education such as universities and colleges that increased from 22% in 2000 to 68% in 2018 (AllahMorad S, 2020).

Our study is limited by some factors. The number of female participants was higher than male, and this does not represent the population distribution of Saudis. As of mid-2020, the male represents 58% of population (Stat.gov.sa, 2020). This owes to the design of study that relies on snowball technique that may impact the sample diversity (Kirchherr J & Charles K, 2018). In addition, the study didn’t specify the use of aspirin based on the “current” and “previous” user. It also didn’t stratified participants based “low-risk” and “high-risk” for CVD. However, our study showed interesting results regarding the prevalence, characteristics, patterns, attitude and knowledge of aspirin use.

Popular use of aspirin as primary prevention agent (n = 341; 44%) among our participants is evident. Almost half of the participants (45%) use their aspirin as primary agent without any advice from healthcare providers. Additionally, around 31% use their aspirin without prescription. Such practice may expose the general population to the harmful adverse effects of aspirin. Therefore, it may become necessary for healthcare authorities in Saudi Arabia to launch educational campaign for both: (1) healthcare providers to direct the practice towards the personalized prescription of aspirin taking into considerations patient’s factors such age, cardiovascular risk, smoking status, obesity and other comorbidities, and (2) community in order to clarify the myth of “very possible benefit of aspirin with lack of any harm” that is circulating among general population.

## 6 Conclusion

Aspirin use is commonly prevalent Saudi population with good level of knowledge of the therapy; however, its popular use as primary preventive agent for CVD may necessitate medical advice based on the level of cardiovascular risk.

## Declaration of Competing Interest

The authors declare that they have no known competing financial interests or personal relationships that could have appeared to influence the work reported in this paper.
